# Direct Detection and Quantification of Bacterial Genes Associated with Inflammation in DNA Isolated from Stool

**DOI:** 10.4236/aim.2014.415117

**Published:** 2014-11

**Authors:** Ramón Gómez-Moreno, Iraida E. Robledo, Abel Baerga-Ortiz

**Affiliations:** 1 Department of Biochemistry, University of Puerto Rico, Medical Sciences Campus, San Juan, Puerto Rico; 2 Molecular Sciences Building, University of Puerto Rico, Medical Sciences Campus, San Juan, Puerto Rico; 3 Department of Microbiology and Medical Zoology, University of Puerto Rico, Medical Sciences Campus, San Juan, Puerto Rico

**Keywords:** Microbiota, Stool Samples, Gut Inflammation, PCR, Microbial Biomarkers

## Abstract

Although predominantly associated with health benefits, the gut microbiota has also been shown to harbor genes that promote inflammation. In this work, we report a method for the direct detection and quantification of these pro-inflammatory bacterial genes by PCR and qPCR in DNA extracted from human stool samples. PCR reactions were performed to detect (i) the *pks* island genes, (ii) *tcpC*, which is present in some strains of *Escherichia coli* and (iii) *gelE* presented in some strains of *Enterococcus faecalis*. Additionally, we screened for the presence of the following genes encoding cyclomodulins that disrupted mammalian cell division: (iv) *cdt* (which encodes the cytolethal distending toxin) and (v) *cnf-*1 (which encodes the cytotoxic necrotizing factor-1). Our results show that 20% of the samples (N = 41) tested positive for detectable amounts of *pks* island genes, whereas 10% of individuals were positive for *tcpC* or *gelE* and only one individual was found to harbor the *cnf-*1 gene. Of the 13 individuals that were positive for at least one of the pro-inflammatory genes, 5 were found to harbor more than one. A quantitative version of the assay, which used real-time PCR, revealed the pro-inflammatory genes to be in high copy numbers: up to 1.3 million copies per mg of feces for the *pks* island genes. Direct detection of specific genes in stool could prove useful toward screening for the presence of pro-inflammatory bacterial genes in individuals with inflammatory bowel diseases or colorectal cancer.

## 1. Introduction

The gut microbiota contains between 5 and 8 million active genes distributed over a great diversity of mostly uncultivable and novel species [1]–[3]. This complex community of bacteria has been shown to promote a healthy gut homeostasis as it is tasked with housekeeping functions like catabolism of complex polysaccharides, biosynthesis of vitamins and other essential nutrients, degradation of xenobiotics and the maintenance of serotonin levels in plasma [4]–[6]. Additionally, it is believed that a healthy commensal gut microbiota aids in the clearance of pathogenic bacteria and in the prevention of intestinal inflammation [7] [8]. Shifts in the composition of the resident microbiota, or microbial dysbioses, have been shown to lead to a variety of intestinal disorders [9] [10]. With the advent of powerful tools for parallel DNA sequencing, there has been a renewed interest in exploring the possible involvement of the gut microbiota in the development of intestinal disorders such as inflammatory bowel Disease (IBD), colorectal cancer (CRC) and gut-brain associated diseases [11]–[13].

Clinical correlations have been established that link chronic bacterial infections and intestinal disorders. For instance, the human pathogen *Helicobacter pylori* is widely recognized as a strong cofactor for the development of gastric adenocarcinoma [14]. Similarly, gut colonization with *Clostridium difficile* is followed by development of colitis, toxic megacolon and perforation [15].

In addition to the known gastrointestinal pathogens, there are the typically non-pathogenic members of the microbiota, which may harbor genes associated with inflammation. For instance, certain strains of commensal *Escherichia coli* have been found to harbor a genomic island encoding a colibactin polyketide synthase multienzyme (*pks* island) that induces DNA damage followed by the activation of DNA repair pathways [16]. The presence of the *pks* island induces mammalian chromosome instability in mouse and human colon cells in culture [17]. Strains of *E. coli* harboring the *pks* island were found in higher frequency in mucosal samples from individuals suffering from CRC and IBD compared with the control group [18].

Other pro-inflammatory gene products found in certain strains of *E. coli* include *tcpC*, which has been found to impair toll-like receptor (TLR) function and the cytotoxic necrotizing factor-1 (CNF-1), an activator of oncogenic Rho GTPases [19]–[21]. Additionally, there are certain strains of commensal *Enterococcus* species that are not pathogenic but contain a subset of the virulence genes including *gelE*—a gene encoding a collagen-degrading metalloprotease which has been found to induce inflammation in a mouse model [22].

It has been established that these pro-inflammatory genes promote the colonization of pathogenic strains of bacteria. Substantially less clear is the effect of these pro-inflammatory genes when harbored by non-pathogenic commensal bacteria in the microbiota over a period of time [23]. Additionally, the distribution and frequency of most of these pro-inflammatory genes in the human population are not known and methods have not been implemented for the quick and direct detection of these genes in easily obtained stool samples.

In this work, we have developed a rapid assay for the detection of pro-inflammatory genes directly in a small stool sample (0.5 g). The assay can be performed in diagnostic format (classical PCR) or a quantitative format (rtPCR). Our results show that pro-inflammatory genes are highly prevalent (up to 20% of the samples tested) and in high enough copy number (up to 1.3 million copies per mg stool) for direct detection in a small stool sample. The assays described in this report could prove useful in future explorations of the relationship between the presence of these pro-inflammatory genes and intestinal diseases.

## 2. Materials and Methods

### 2.1. Stool Samples

Anonymous human stool samples were donated by a clinical laboratory (Laboratorio Clínico El Monte, San Juan, Puerto Rico, N = 41). Samples were stored at −80°C.

### 2.2. DNA Extraction

Bacterial DNA was extracted from 41 human stool samples using the QIAamp^®^ DNA Stool Mini Kit (QIAGEN). Approximately 0.2 g stool was resuspended in 1.4 mL of ASL buffer. The bacterial DNA was extracted following the manufacturer’s protocol and eluted in 200 μl of the buffer included in the kit. A total of 5 μl of DNA extract was used as template for the ensuing PCR reactions.

### 2.3. Detection of Bacterial Genes by PCR

A volume of 5 ul of undiluted fecal DNA extract (from 0.2 mg stool) was used and the PCR conditions are summarized in Table 1. All PCR reactions begin with an initial denaturation of 1 minute at 94 C followed by 30 cycles of denaturation (30 seconds at 94°C), annealing (30 seconds at different temperatures shown in Table 1) and extension (3 minutes at 68°C). All reactions were finalized with a long extension step of 10 minutes at 72°C to ensure maximum detection. The detection of *pks island* genes was performed using the primer for the amplification of *clbN* genes that were described previously [23]. The detection of *gelE* was carried out using gelE-specific primers that were also described previously [24]. For the detection of *tcpC* and *cnf-*1, oligonucleotide primers were designed using the software Primer 3 (http://simgene.com/Primer3) on the nucelotide sequences for *E. coli* ECOR63 (GenBank: GQ903014.1) and *E. coli* MS 153-1 (NCBI: ZP_16446903.1). For the detection of *cdt*, primers were designed using the *cdtB* subunit from *Campylobacter jejuni subsp. jejuni* 81–176 (GenBank: AAB06708.1).

### 2.4. Purification of PCR Products

The PCR products were separated on a 2% agarose gel and extracted from the agarose gel using the QIAquick Gel Extraction Kit (QIAGEN).

### 2.5. DNA Sequence Confirmation by the Sanger Method

The DNA sequence for the amplified fragments was verified using the Sanger method for DNA sequencing at the RCMI Center for Genomics in Health Disparities and Rare Diseases at the University of Puerto Rico. The purified amplicon was sent for sequencing using the forward oligonucleotide primer for each of the products.

### 2.6. Estimation of Gene Copy Number by Quantitative PCR (qPCR)

In order to provide a quantitative estimate on the number of copies of each of the pro-inflammatory genes, we measured the PCR reaction in real time and compared the results for the DNA extracted from stool samples with known amounts of DNA extracted from isolated bacteria known to contain the genes. The qPCR reaction was performed with 6.8 μl of stool DNA extract and the QuantiTect SYBR Green PCR kit (Qiagen) and was read on a Eppendorf Mastercycler Realplex.

### 2.7. Calibration Curve

A calibration curve was built for each gene by performing real-time PCR on the DNA extracts from a bacterial isolate known to contain at least a copy of the bacterial gene of interest. In order to identify strains that could serve as positive controls (or method calibrants) for the *pks* island, *tcpC*, and *cnf* genes, we screened a collection of clinical isolates of *E. coli* that were part of a nosocomial infection surveillance study [25] and found several *E. coli* isolates that tested positive for one of the genes. The DNA extracted from these strains was used as positive control in the PCR reaction, which was done in triplicate. The positive control for *gelE* was *Enterococcus faecalis* strain H32, an environmental isolate known to contain the *gelE* virulence gene and kindly donated by Dr. Luis Ríos-Hernández from University of Puerto Rico, Mayagüez Campus. The genome of *E. coli* contains 4.6 million base pairs and weighs 5.1 × 10^−15^ g. The genome of *E. faecalis*, somewhat smaller, weighs 3.6 × 10^−15^ g. Thus, the DNA copy number conversion can be determined from a measurement of absorbance at 260nm according to the following relationship:

(1) $$\#\text{DNA}\;\text{copies}/\mu l=A260*(50\times 10^{-9}\;g\;\text{DNA}/\mu l)/(\text{GW})$$

where A260 is the absorbance of the sample at 260 nm and GW is the weight in grams of the entire genome of the organism (5.1 × 10^−15^ g for *E. coli* and 3.6 × 10^−15^ g for *E. faecalis*).

For example, the *pks* island gene standards were made with known amounts of *E. coli* DNA. Samples containing 384 ng/μl (75,294,120 copies per μl), 100 ng/μl (19,607,843 copies per μl), 10 ng/μl (1,960,784 copies per μl), 1 ng/μl (196,078 copies per μl) and 0.1 ng/μl (19,607 copies per μl) were used as templates of the qPCR reaction monitored in real time.

The correlation coeffcient (*χ*^2^) for each calibration curve was determined by the least squares method and the percentage efficiency of the PCR reaction calculated by implementing the equation:

(2) $$\text{Efficiency}=-1+10^{\left(-1/\text{slope}\right)}$$

Typical positive values for the *pks* island range between 2800 – 1,300,000 copies per μl.

## 3. Results

### 3.1. Detection and Prevalence of Pro-Inflammatory Genes in Stool Samples

**Figure 1:**
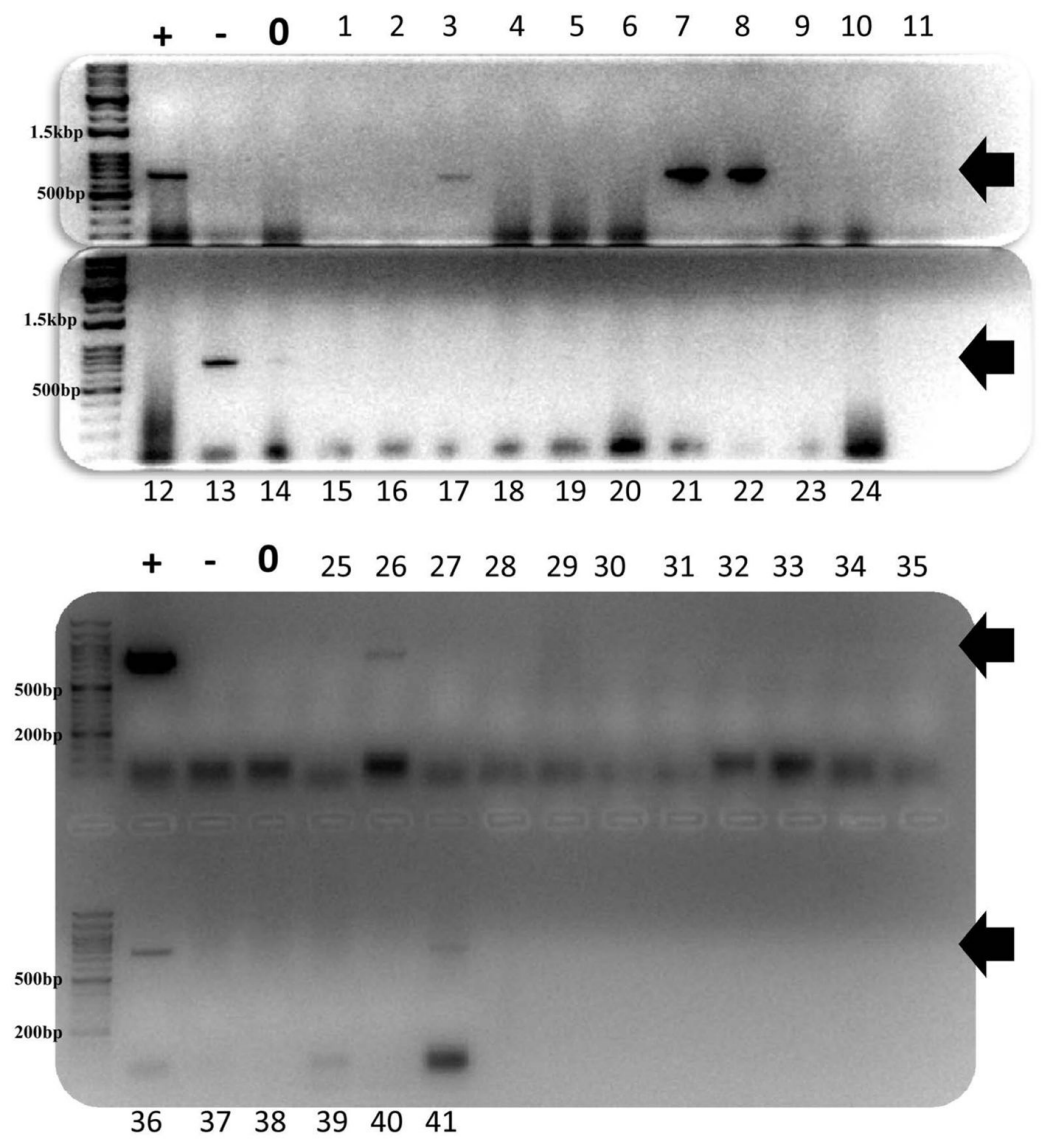
PCR amplification of the *pks* island genes was visualized on a 2% agarose gel in which the first four lanes contain the DNA size marker, a positive control (+), a negative control (−) and a blank (0), respectively. The DNA size markers used for samples 1 – 24 is 1kb Opti-DNA Marker from ABM band for sample 25 – 41 the 100 bp Opti-DNA Marker by ABM. The presence of the *pks* island gene can be established by the presence of a band at 733 bp (black arrows). The data for the 41 samples is shown in two different gels with eight positives (3, 7, 8, 13, 14, 26, 36 and 41).

A total of 41 anonymous human stool samples were analyzed for the presence of the genes for *pks* island, *tcpC*, *gelE*, and *cnf-1*. The presence of bacteria was confirmed in all of the samples by amplification of the 16S-rRNA gene (data not shown). The presence of the *pks* island genes was established by the amplification of a DNA fragment of 733 bp (Figure 1(a)). A total of 8 samples out of 41 were found to contain the gene for *pks* island (20%). The other pro-inflammatory gene, *tcpC*, was detected in a total of 7 samples (17%) as evidenced by the presence of a band of 216 bp (Figure 1(b)) and the enterococcal *gelE* was found in 3 samples easily detected by a band of 213 bp (7%). The gene for cytotoxic necrotizing factor-1 (*cnf-*1) was only found in a single sample while the gene for the cytolethal distending toxin (*cdt*) was not found in this group of samples. The results from the PCR screens are summarized in Table 2.

A total of thirteen (13) samples were found to contain at least one of the four pro-inflammatory genes. Of these 13 samples, a total of eight (8) samples were found to contain only one of the genes and four samples were found to contain two pro-inflammatory genes. One result, corresponding to anonymous sample “Hu037” was found to contain three genes: *pks* island, *tcpC* and *gelE* (Table 3). Future work will be aimed at establishing whether these individuals harboring multiple pro-inflammatory bacterial genes have a higher risk of developing intestinal disorders.

**Figure 2:**
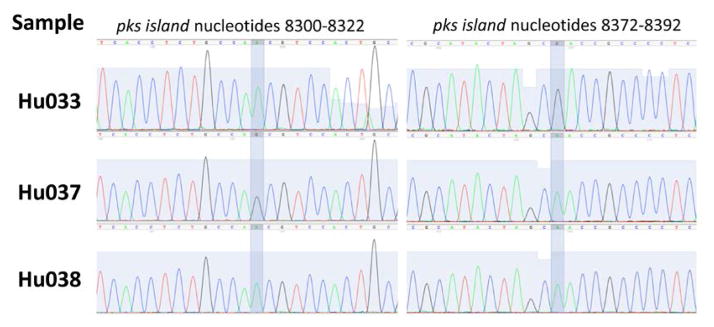
The DNA sequences for all the fragments generated in this study were confirmed by the Sanger method. Interestingly, three distinct variants of the *pks* island sequence were identified in three different samples (Hu033, Hu037 and Hu038). The sequencing chromatograms corresponding to regions 8300 – 8322 and 8372 – 8392 of the *pks* island gene sequence (Ge-neBank Accession No. AM229678) are shown for all three samples. Sequence variability was observed in positions 8312 and 8383 of this amplicon.

The DNA sequences for the PCR amplified pro-inflammatory bacterial genes were confirmed by performing Sanger sequencing on the gel-purified PCR products. By comparing the DNA sequences found in individual samples, we were able to make a preliminary assessment of the sequence variability for the pro-inflammatory bacterial genes. While the DNA sequences for the amplicons generated from *tcpC* and *gelE* were exactly the same in all positive stool samples, the DNA sequence for the *pks* island amplicon showed variability around sequence positions 8312 and 8383 (numbering according to GeneBank accession No. AM229678). The amplicon generated from the *pks* island in samples Hu033, Hu037, Hu038 contains three distinct variants (Figure 2). Stool sample labeled Hu033 features A and G in positions 8312 and 8383, respectively. Meanwhile, the stool samples Hu037 and Hu038 have a G-A and A-A in those positions.

### 3.2. Quantification of Pro-Inflammatory Genes in Stool

**Figure 3:**
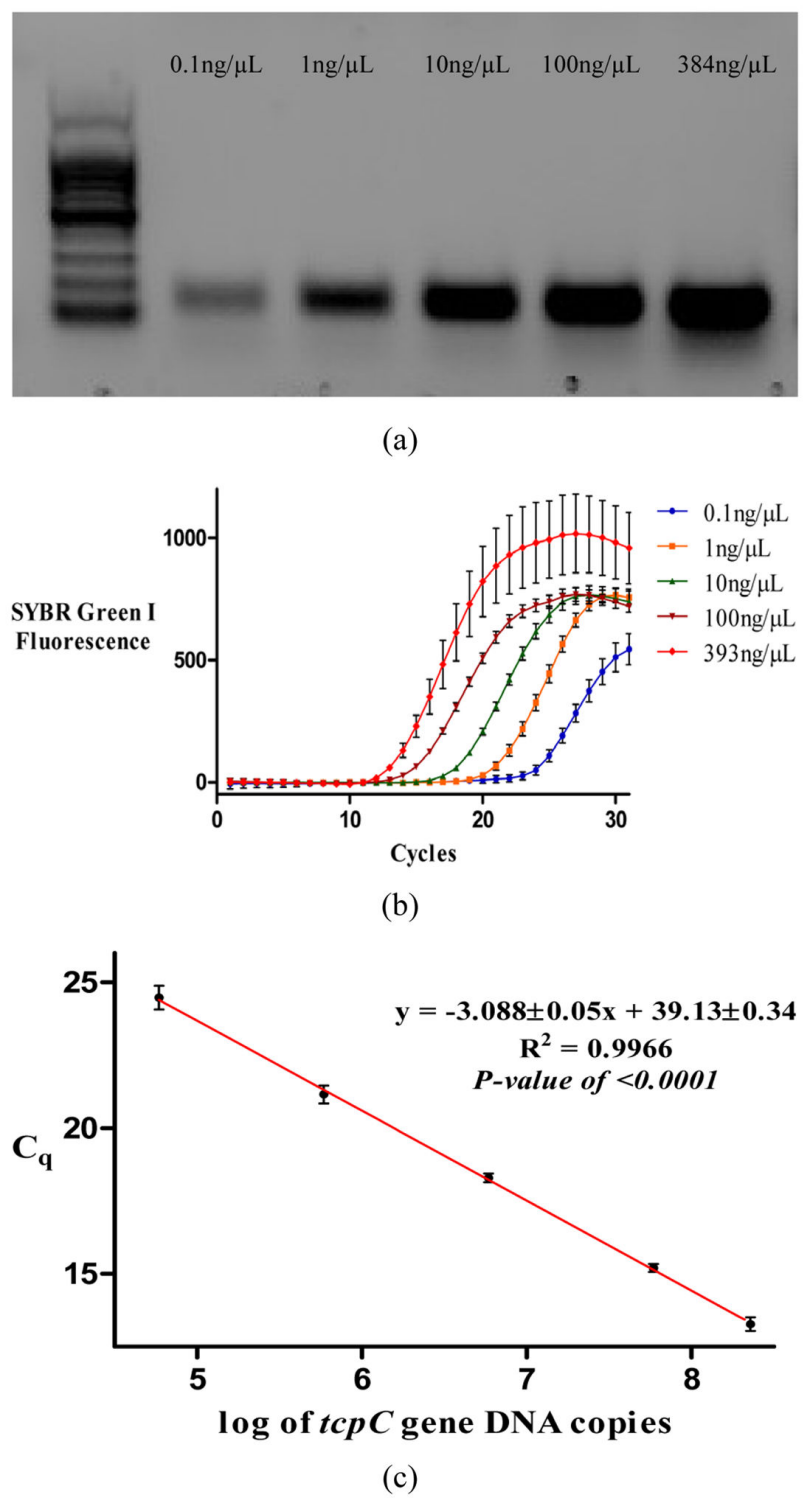
For the quantification of DNA copy number, a standard curve was generated for each of the pro-inflammatory genes as described in the methods section. Standards containing 393 ng/μl (77,058,825 copies per μl), 100 ng/μl (19,607,843 copies per μl), 10 ng/μl (1,960,784 copies per μl), 1 ng/μl (196,078 copies per μl) and 0.1 ng/μl (19,607 copies per μl) were used to make the calibration curve. Typical copy number estimates for *tcpC-*positive samples ranged from 100,000 – 800,000 copies per μl. In this figure we show (a) the agarose gel electrophoresis for the PCR amplification of *tcpC* gene using different known amounts of *E. coli* DNA in the reaction mixture, (b) the qPCR reaction for the standards monitored in real time and (c) the calibration curve for the quantification of *tcpC* copies.

From the PCR results visualized on an agarose gel, it was clear that some samples registered a more intense band than others, raising the possibility that some individuals harbor a higher content of bacterial pro-inflammatory genes than others. In order to provide a quantitative estimate of the number of copies of the pro-inflammatory genes per patient, we performed the PCR assay with real-time detection (Figure 3). A standard curve that was made with known amounts of DNA copies as described in the methods section, enabled the quantification of DNA copy number for each of the genes in the 41 stool samples. The number of copies of *pks* island among those who tested positive fluctuated between 2800 and 1.3 million copies per mg of stool analyzed and the number of *gelE* copies fluctuated between 6000 – 260,000 copies per mg of stool. The amount of *tcpC* DNA was less variable fluctuating between 100,000 – 800,000 copies per mg of stool. Future work will be aimed at establishing whether a correlation exists between a high copy number for these pro-inflammatory genes and a higher risk of developing gastrointestinal disorders.

## 4. Discussion

The elucidation of the role of the gut microbiota in human health is an active area of research [26]–[28]. A number of efforts worldwide have served to elucidate important associations between the microbiota and health [29]–[31]. For instance, an unequivocal link has been established between the gut microbiota and obesity [32] [33]. Additionally, the presence of pathogens such as *Helicobacter pylori* and *Clostridium difficile* has been found to correlate with inflammation and ultimately with the development of gastric cancer [14] [15]. More recently, there have been reports that the gram-negative bacterium of the genus *Fusobacterium* is more highly represented in colorectal tumor tissue than in normal mucosal tissue from the same patients [34] [35].

In this report, instead of focusing on the presence of bacterial species, we have developed a quantitative assay to detect specific bacterial genes that have been associated with inflammation, directly in a small stool sample. Some of these genes in this report were found to be present in as many as 20% of the stool samples and in copy numbers as high as 1.3 million copies per mg of stool. The assay described in this report provides a quick method for the screening of stool samples to ascertain the presence of pro-inflammatory genes in case-control studies involving individuals with colorectal cancer or inflammatory bowel diseases. The genes in this report, namely *pks* island, *tcpC*, *cnf-*1, *gelE* and *cdt*, have all been the subject of scrutiny for their possible crosstalk with inflammatory processes. Here we discuss each of them separately:

### *pks* Island

Of the genes tested, the *pks* island was the most prevalent and was found in high copy numbers (>10^6^ copies per mg stool). The *pks* genomic island encodes a number of multidomain enzymes termed polyketide synthases that typically synthesize secondary metabolites in numerous microbial species, although the actual compound made by this multienzyme has not been chemically characterized [16]. The *pks* island has been found in pathogenic and in non-pathogenic strains of *E. coli* [23]. Although the *pks* island is not pro-inflammatory per se, the presence of this genomic island in some strains of *E. coli* has been implicated in the formation of colorectal tumors in inflammation-prone mice, which lack the inteleukin-10 gene [18]. That study also reported that the *pks* island was more highly represented in the intestinal mucosa from colorectal cancer (CRC) patients than in non-CRC controls [18]. In the present study, the *pks* island genes were found in 20% of the stool samples analyzed, a frequency consistent with previous findings reporting the *pks* island to be present in 20% of strains isolated from human mucosa samples from non-cancer donors [18] and in 32% of *E. coli* strains isolated from the feces of hospitalized veterans [23]. Although our assay is carried out on total DNA isolated from a stool sample, without strain isolation or cultivation, our results are similar to those obtained using the more conventional culture-dependent method.

### tcpC

Similarly, the *tcpC* gene has been found in strains of *E. coli* associated with severe kidney infection [36]. *tcpC* subverts toll-like receptor-dependent inflammation by binding directly to the receptor, resulting in the formation of kidney abscesses in mice infected with strains of uropathogenic *E. coli* [19]. However, there are no reports of the *tcpC* gene in the commensal *E. coli* strains that inhabit the GI tract. Much to our surprise, seven (7) of our fecal samples were found to harbor the tcpC gene in high enough copy number to detect by PCR.

### cnf-1

The *cnf-*1 gene encodes an enzyme, the cytotoxic necrotizing factor-1, which is found in strains of *E. coli* commonly associated with urinary tract infections [37]. The Cnf enzyme deaminates Rho GTPase in glutamine 63 causing it to be locked in the active GTP-bound state. Although the *cnf-*1 gene has been found in bacterial isolates from feces of children with diarrhea, this is the first report of this gene in high enough copies to be detected directly in stool [38]. Only one (1) sample in the present study was found to contain the *cnf-*1 gene.

### gelE

*GelE* gene encodes a metallo-collagenase from the genus *Enterococcus*. The GelE collagenase has been found to promote chronic inflammation in mice lacking the *interleukin-*10 gene [22]. More recently, *gelE* gene was found in higher frequency in bacterial isolates from the feces of children with IBD vs. children without IBD [39]. Here we report 3 stool samples with detectable levels of *gelE* gene directly in stool.

With the growth of affordable parallel sequencing methods, scientists have gained a whole new view of the diversity of bacterial species (or taxa) in a complex community such as the gut microbiota [40] [41]. One important line of experimentation centers on building a comprehensive list of bacterial species present in the community for comparison between disease cases and controls. However, most community profiling efforts rely on molecular markers (such as the 16S rRNA gene) and do not necessarily detect additional genes that may have been acquired in time. For instance, the bacterial community profile for any stool sample would likely reveal the presence of *E. coli*, but it would not necessarily show whether the *E. coli* strains in the sample contain pro-inflammatory genes (*tcpC*, *pks* island, etc.). In addition, *E. coli* is not the only species that harbor the *pks* island, as the genes can also be carried by other bacteria, such as *Klebsiella pneumoniae*, *Enterobacter aerogenes* and *Ci-trobacter koseri* [42].

The assay described in this report is meant to complement ongoing efforts, whether by bacterial community profiling or by shotgun metagenomics, to ascertain the presence of harmful or protective genes, in fecal samples regardless of which species carries the genes. Clearly, other complementary assays will have to be developed to determine whether the pro-inflammatory bacterial genes are being transcribed in the gut, or whether the gene products are being synthesized in active form. But the development of a quick and quantitative assay for the detection of pro-inflammatory genes in stool samples is a first step toward the quick interrogation of stool samples in large-scale case-control studies involving IBD and CRC patients.

## 5. Conclusion

We report the detection, directly in DNA extracted from a fecal sample, of several genes of bacterial origin which had been previously associated with inflammation. Although the presence of these genes had been ascertained in strains of bacteria known to colonize other tissues, this is the first report of these genes being found in the normal commensal flora. The assay described in this report provides a quick and inexpensive method for the analysis of a large number of samples from case-control studies involving patients of IBD or CRC.

## Figures and Tables

**Table 1:** Summary of PCR conditions for the detection of a panel of pro-inflammatory bacterial genes.

Bacterial Gene	Annealing Temp	Primer Sequences (5′ to 3′)	Product Size (bp)
*pks* island	63°C	F: TCGATATAGTCACGCCACCA R: GTCAAGCGAGCATACGAACA	733
*tcpC*	56°C	F: TCGGCGATAGCTTAAGGAGA R: CCGCCAAATAATGGCTGTAT	216
*gelE* [24]	56°C	F: TATGACAATGCTTTTTGGGAT R: AGATGCACCCGAAATAATATA	213
*cnf-*1	56°C	F: AGCGTGCAATCTATCCGTATTT R: TGGAATTTCCCCAGTATAGGTG	173
*cdt*	50°C	F: AGATATCTTAATGATACAAGAAGCA R: TGAAACTATAGCTAAATTTACACG	196

**Table 2:** Summary of the results from the PCR screening of pro-inflammatory bacterial genes from anonymous stool samples (N = 41). A DNA calibration curve was made by real-time PCR measurements of samples with known DNA concentration and each point was measured in triplicate.

Bacterial Gene	# Positives	%	Calibration Curve (*χ*^2^)	Efficiency (%)	Copy Number in Positives per mg Stool
*pks* island	8	20	0.9991	114.092	2800 – 1,300,000
*tcpC*	7	17	0.9966	99.09	100,000 – 800,000
*gelE*	3	7	0.9875	58.75	6000 – 260,000
*cnf-*1	1	2	0.9987	104.69	43,000
*cdt*	0	0	N/A	N/A	N/A

**Table 3:** Stools samples positive for pro-inflammatory bacterial genes.

Samples	*pks* Island	*tcpC*	*gelE*	*cnf-1*	*cdt*
HUO32	+	−	−	−	−
HUO37	+	+	+	−	−
HUO38	+	−	−	−	−
HUO40	−	+	−	−	−
HUO33	+	+	−	−	−
HUO47	+	−	−	−	−
PR014	−	−	+	−	−
LEMB3_2	+	+	−	−	−
LEMB3_6	−	−	+	−	−
LEMB3_8	−	+	−	−	−
LEMB3_12	+	−	−	+	−
LEMB3_15	−	+	−	−	−
LEMB3_17	+	+	−	−	−
